# Роль цитокинов в процессах адаптивной интеграции иммунных и нейроэндокринных реакций организма

**DOI:** 10.14341/probl12744

**Published:** 2021-04-08

**Authors:** Е. А. Трошина

**Affiliations:** Национальный медицинский исследовательский центр эндокринологии

**Keywords:** цитокины, эндокринная система, иммунная система, нервная система, аллостаз, интерлейкины

## Abstract

Иммунная, эндокринная и нервная системы интегрированы благодаря существованию взаимных путей передачи информации об изменениях их фактического функционального состояния. Главными задачами мозга являются прием, интеграция и хранение информации, и есть убедительные доказательства, что это относится и к информации, полученной с помощью иммунных реакций организма. Доказано, что выработка цитокинов в головном мозге может быть вызвана не только периферической иммунной стимуляцией, но и собственно нервными клетками, стимулированными определенными нейросенсорными сигналами. Эволюционно сохраненные антигомеостатические механизмы, характерные для конкретных заболеваний, — предмет дальнейших исследований, результаты которых могут быть очень важны для разработки терапевтических стратегий, которые препятствовали бы нежелательным комбинированным эффектам иммунных и нейроэндокринных медиаторов.

## Abstract (en)

The immune, endocrine and nervous systems are integrated due to the existence of reciprocal pathways for transmitting information about changes in their actual functional state. The main task of the brain is to receive, integrate and store information, and there is strong evidence that this also applies to information obtained through the body’s immune responses. It has been proven that the production of cytokines in the brain can be caused not only by peripheral immune stimulation, but also by the nerve cells themselves, stimulated by certain neurosensory signals. Evolutionarily preserved antihomeostatic mechanisms characteristic of specific diseases are the subject of further research, the results of which may be very important for the development of therapeutic strategies that would prevent the undesirable combined effects of immune and neuroendocrine mediators.

Иммунная, эндокринная и нервная системы интегрированы благодаря существованию взаимных путей передачи информации об изменениях их фактического функционального состояния. Главными задачами мозга являются прием, интеграция и хранение информации, и есть убедительные доказательства, что это относится и к информации, полученной с помощью иммунных реакций организма.

Известно, что мозг объединяет все виды сенсорной информации, множество данных подтверждает наличие двунаправленной связи между нейроэндокринной и иммунной системами на протяжении всех этапов жизни человека. Возможность обработки и интеграции с прочими сигналами информации, сопряженной с функционированием системы иммунитета, представляет собой феномен биологической целесообразности на уровне центральной нервной системы человека. Не исключено, что он отчасти может быть сопряжен с системой цитокинового участия в формировании так называемой синаптической пластичности.

Доказано, что выработка цитокинов в головном мозге может быть вызвана не только периферической иммунной стимуляцией, но и собственно нервными клетками, стимулированными определенными нейросенсорными сигналами. Например, психосенсорный стресс индуцирует прямую церебральную продукцию цитокинов [1].

В принципе, выработка иммунорегуляторных цитокинов в головном мозге — это физиологический процесс, который имеет важнейшее значение для реализации основных функций мозга, например, памяти, обучения, адаптации. Этот процесс объясняется биологическими свойствами цитокинов, которые представляют собой молекулы, обеспечивающие взаимодействие клеток посредством множества различных механизмов, а именно:

паракринное (воздействие через растворимые медиаторы на соседние клетки); матрикринное (цитокины в неактивной форме связываются с протеогликанами межклеточного матрикса и высвобождаются под действием металлопротеиназ); юкстакринное (сигнал передается через плазматическую мембрану соседней клетки), эндокринное (системное действие цитокинов, поступающих в кровоток).

Источниками продукции цитокинов являются активированные Т- и В-лимфоциты, макрофаги, а также (в ряде случаев) клетки, которые не имеют отношения к иммунной системе (например, интерлейкин (ИЛ)-6 вырабатывают фолликулярно-звездчатые клетки гипофиза, эндотелиальные и синовиальные клетки, хондроциты и др.)

Различают провоспалительные и противовоспалительные цитокины (ИЛ-2, ИЛ-10, ИЛ-27, ИЛ-35 и ИЛ-37), а также обладающие двоякими свойствами (например, ИЛ-6). Кроме того, выделяют регуляторные цитокины, которые обеспечивают толерантность к собственным тканям, минимизацию тканевого повреждения. Координированная выработка элементами иммунной системы различных цитокинов происходит в непосредственной близости к клеткам-мишеням.

Источники продукции и функции некоторых цитокинов представлены в таблице.

**Table:** Таблица. Цитокины, источники их продукции и функции [2]

ИЛ-2	T-клетки, NKT, ДК, ТК	Стимулирует ответ Th1 и Th2, ингибирует ответ Th17 и дифференцировку Tfh. Стимулирует развитие Treg, B-клеток, моноцитов, NKT
ИЛ-9	T-клетки, ТК	Стимулирует развитие Th17, B-клеток
ИЛ-15	Моноциты, макрофаги, ДК	Стимулирует пролиферацию T-клеток и ЕК. Защищает нейтрофилы от апоптоза, модулирует фагоцитоз и стимулирует секрецию ИЛ-8. Индуцирует пролиферацию и дифференцировку В-клеток, усиливает синтез Ig плазмоцитами
ИЛ-21	CD4+, преимущественно Tfh	Стимулирует пролиферацию и действие CD4+ и CD8+, негативно регулирует дифференцировку и активность Treg. Стимулирует дифференцировку B-лимфоцитов в клетки памяти, усиливает синтез Ig плазмоцитами. Увеличивает активность макрофагов и ЕК
ИЛ-4	T-клетки, ТК, базофилы, эозинофилы	Усиливает ответ Th2, ингибирует дифференцировку Th1 и Th17. Регулирует альтернативный путь активации макрофагов. Активирует В-клетки и индуцирует экспрессию МНС II (главный комплекс гистосовместимости II класса) на покоящихся В-клетках
ИЛ-13	T-клетки, ТК, базофилы, эозинофилы, NKT, моноциты, макрофаги, ДК	Усиливает ответ Th2. Стимулирует созревание и дифференцировку В-клеток. Ингибирует продукцию провоспалительных цитокинов
ИЛ-7	B- и T-клетки	Индуцирует ответ Th1 и Th17. Стимулирует рост пре- и про-В-клеток
ИЛ-6	B- и T-клетки, моноциты	Индуцирует острофазный ответ совместно с ИЛ-1 и ФНОα. Дифференцировка В-клеток в плазмоциты, усиление активности плазмоцитов. Дифференцировка моноцитов и Т-клеток
ИЛ-12	Активированные фагоциты и ДК	Дифференцировка наивных Т-клеток в Th1, стимуляция продукции ИФНγ и ФНОα ЕK и NKT, усиление цитотоксической активности ЕК и СD8+
ИЛ-23	Активированные ДК и фагоциты	Совместно с ИЛ-6 и TGF-β стимулирует дифференцировку наивных CD4+ в Th17
ИЛ-35	Стимулированные Treg	Противовоспалительный/иммуносупрессивный цитокин
Семейство цитокинов ИЛ-1	Продуцируются многими клетками, но в особенно большом количестве — моноцитами, макрофагами, ДК	Провоспалительные: ИЛ-1А, ИЛ-1В, ИЛ-18, ИЛ-36А, ИЛ-36В, ИЛ-36G, ИЛ-33. Активируют врожденный и приобретенный иммунитет, острое и хроническое воспаление. Противовоспалительные: ИЛ-1Ra, ИЛ-36Ra, ИЛ-37
Семейство ИЛ-17 (ИЛ-17A, ИЛ-17B, ИЛ-25, др.)	Th17, CD8+ T-клетки, ЕК, NKT, нейтрофилы	Активация транскрипционных факторов, индуцирующих экспрессию генов антибактериальных пептидов, провоспалительных хемокинов и цитокинов и металлопротеиназ
ИЛ-10	T-клетки, B-клетки, моноциты, макрофаги, ДК	Иммуносупрессивное действие
ИЛ-8	Моноциты, макрофаги, нейтрофилы, лимфоциты	Хемоаттрактант для нейтрофилов, ЕК, T-клеток, базофилов и эозинофилов
ИЛ-5	Th2, активированные эозинофилы, ТК, CD8+ Т-клетки, ЕК, NKT	Дифференцировка и функционирование миелоидных клеток; усиление хемотаксической активности и способности к адгезии эозинофилов; заживление ран
ИЛ-21	T-клетки	Дифференцировка, пролиферация и функционирование CD8+, Тh17, Тreg, Tfh и ЕК, активация В-клеток и дифференцировка зрелых и В клеток памяти в плазматические клетки
ФНОα	Активированные макрофаги, моноциты, CD4+ Т-клетки, В-клетки, ЕК, нейтрофилы, ТК	Провоспалительный цитокин, который индуцирует острофазные белки и стимулирует ангиогенез
Лимфотоксин-α	Th1, CD8+ Т-клетки, ЕК, В-клетки, макрофаги	Член семейства ФНО, который участвует в регуляции гомеостаза ДК и CD4+ Т-лимфоцитов, а также оказывает прямой цитотоксический эффект на опухолевые клетки
TGF-β	Эозинофилы, макрофаги, Treg	Иммуносупрессивное действие, влияет на генерацию Treg и толерогенных ДК, существенно снижает цитотоксическую и цитокинпродуцирующую активность моноцитов/макрофагов, снижает продукцию ими нитросоединений, реакционно-способных радикалов и провоспалительных цитокинов

Примечание. Th — Т-хелпер; Treg — регуляторные T-лимфоциты; ДК — дендритные клетки; ЕК — естественные киллеры; ТК — тучные клетки; ИФН — интерферон; ФНО — фактор некроза опухоли; АПК — антигенпрезентирующие клетки; Tfh — фолликулярные Т-хелперы; NKT — естественные Т-клетки; FoB — фолликулярные В-лимфоциты; Breg — регуляторные В-лимфоциты; СD — кластеры дифференцировки (Clusters of Differentiation); ИЛ — интерлейкин; Ig — иммуноглобулин.

На сегодняшний день выявлена продукция ряда цитокинов в центральной нервной системе (ЦНС) [3], а введение, например, ИЛ-18 индуцирует экспрессию его собственного гена в головном мозге [4]. Есть данные о том, что ИЛ-1 и ИЛ-6 вырабатываются непосредственно в гипоталамусе и гипофизе во время периферических специфических иммунных реакций. Индуцированные таким образом цитокины важны для синаптической пластичности, так, ИЛ-1 поддерживает, а ИЛ-6 ингибирует механизмы, ответственные за обучение и консолидацию памяти [3][5][6].

Существует теория, что физиологические воздействия на функции мозга тех цитокинов, которые продуцируются вслед за периферическими иммунными или центральными нейрональными сигналами, следует рассматривать в контексте общепринятой концепции тройственного синапса, включающей астроциты [7] в качестве третьей стороны. Есть данные, что астроциты как нейроиммунные клетки, взаимодействующие с клетками микроглии, могут играть центральную роль в интеграции иммунных сигналов в мозг с сохраненной или недавно полученной информацией от других высокоспециализированных сенсорных систем.

Ранее считалось, что астроциты играют лишь вспомогательную роль в нейронной активности, но сегодня известно, что благодаря своему широкому распространению и тесному контакту с нейронами эти клетки представляют собой основные компоненты нейрональной среды и микроархитектуры паренхимы головного мозга, а также хранят и обеспечивают энергетические субстраты, контролируют развитие нейронных клеток, синаптогенез и синаптическую активность. Благодаря своим иммунным функциям астроциты являются частью внутренней защитной системы мозга [8]. Следовательно, они из-за их двойственных нейронных и иммунных функций могут быть расценены как «нейроиммунные» клетки, причем их функции включают в себя выработку различных медиаторов с иммунными и нервными эффектами, таких как ИЛ-1, ИЛ-6 [9].

Трехсторонний синапс может играть центральную роль в обработке иммунных сигналов в головном мозге и в их интеграции с нейросенсорными сигналами и обеспечивает молекулярные и клеточные основы для обработки крайне изменчивого массива подобных сигналов [3].

Итоговое влияние на механизмы мозга и на нейроэндокринную иммунорегуляцию тех цитокинов, которые вырабатываются при активации тройственного синапса, будет зависеть от того, как, когда и где происходит такая стимуляция, а также от типа задействованного синапса. Например, когда активируется повышенная продукция ИЛ-1 и других цитокинов, их воздействие на тройственный синапс будет заключаться в модуляции физиологических функций мозга. Когда их продукция в головном мозге будет иммунологически инициирована, их действие будет преобладать в областях мозга, в которых эти медиаторы могут восстанавливать гомеостаз и оказывать иммунорегуляторное действие, вызывая нейроэндокринные реакции.

Когда иммунные эффекты не ведут к повреждению собственных компонентов, они обычно рассматриваются как защитные адаптивные механизмы. В целом иммунные реакции можно рассматривать как гомеостатические, обеспечивающие поддержание постоянства молекулярных и клеточных компонентов организма, для обеспечения данного эффекта нужно добиться биологической стабильности посредством физиологических или поведенческих изменений. Однако данный процесс при длительном его поддержании имеет так называемую «функциональную стоимость», или аллостатическую нагрузку, и может способствовать проявлению хронических заболеваний [10].

Понятие аллостаза обычно связывают с реакциями на стресс, следовательно, и иммунный стресс может играть определенную роль, так как активность большинства физиологических систем должна быть скорректирована для мобилизации иммунных клеток. Нейроэндокринные реакции в сочетании с иммунными опосредуют метаболические изменения и обеспечивают появление иммунорегуляторных сигналов, оказывая определенное противодействие регуляторным гомеостатическим механизмам, которые стремятся поддерживать систему на заданном уровне регуляции. В свою очередь, это является физиологически затратным, особенно при процессах, основанных на резком повышении активности иммунной системы [11]. Предполагается, что физиологически регулирование затрат энергии должно быть приведено в соответствие с новым заданным значением, причем процесс должен быть обратимым при восстановлении прежних условий. Если же такая обратимость нарушается во время длительных ситуаций, вновь заданные условия способствуют манифестации патологических состояний, в т.ч. аутоиммунных заболеваний.

Тем не менее иммунные реакции способствуют и восстановлению гомеостаза путем высвобождения цитокинов и других продуктов, которые, помимо своих собственных иммунных функций, могут также индуцировать нейроэндокринные реакции, оказывающие влияние на поддержание защитных сил организма. Важную роль в этом играет гипоталамо-гипофизарно-надпочечниковая (ГГН) система. Доказано, что ИЛ-1 и ИЛ-6, вырабатываемые воспалительными клетками, являются стимуляторами эндокринной системы посредством синтеза адренокортикотропного гормона (АКТГ). Данный импульс, по-видимому, проходит по гипоталамическим рецепторам, вследствие чего ЦНС вступает во взаимодействие с эндокринной и иммунной системами в ответ на воздействие патогенов. Более того, данная связь свидетельствует о том, что регуляция ГГН-оси цитокинами при воспалении зависит от АКТГ, но, как показано в ряде исследований, длительная стимуляция с помощью ИЛ-6 не гарантирует устойчивого повышения уровня АКТГ.

Так или иначе, в норме кортизол регулирует уровни нескольких циркулирующих в крови провоспалительных цитокинов, таких как ИЛ-2, ИЛ-3, ИЛ-6, фактор некроза опухоли альфа (ФНО-a) и интерферон-γ (ИФН-γ), и влияет на активность и жизнеспособность клеток иммунной системы, угнетает фагоцитоз антигенов и их последующую элиминацию макрофагами. Кортизол угнетает как клеточный, так гуморальный иммунный ответ, поддерживая баланс про- и противовоспалительных реакций, и вызывают инволюцию лимфоидных органов, снижает фагоцитарную активность нейтрофилов и макрофагов, подавляет активность лимфоцитов, тормозя их созревание и дифференцировку, стимулируя апоптоз. За счет иммуносупрессивного эффекта — снижает количество и активность воспалительных клеток, особенно тканевых макрофагов, и ограничивает их способность реагировать на поступающие антигены. Подавление активности иммунных клеток нарушает их дегрануляцию и высвобождение разрушающих ткани ферментов (матриксных металлопротеиназ, протеаз, нуклеаз и др.), хемоаттрактантов, адгезивных молекул.

У T-лимфоцитов кортизол снижает генетическую экспрессию ИЛ-2, подавляет работу Т-клеточного рецептора и молекул, следующих за ним по сигнальному пути (лимфоцитоспецифической протеинтирозинкиназы, инозитол-1,4,5-трифосфата), посредством мембраносвязанных рецепторов. Помимо этого, кортизол вызывает повышенную экспрессию высокоаффинного рецептора ИЛ-2 на регуляторных Т-клетках.

Показано, что глюкокортикоиды (ГК) снижают выработку цитокинов, хемокинов, производных арахидоновой кислоты, количество базофилов, а также выработку гистамина. Каким образом ГК влияют на гуморальный иммунитет, до конца не определено. Однако они, бесспорно, эффективны в лечении целого ряда аутоиммунных заболеваний, развивающихся при участии антител. Известно, что В-лимфоциты обладают мощными иммунорегуляторными функциями, особенно через экспрессию ИЛ-10, а ген ИЛ-10, в свою очередь, известен как мишень глюкокортикостероидов (ГКС) в макрофагах. Несмотря на это, механизмы взаимодействия между сигнальными путями рецепторов ГКС и рецепторов В-лимфоцитов пока недостаточно изучены [2].

В целом подавление иммунного ответа под действием кортизола связано с ослаблением процессинга антигенов, снижением выработки антител, нарушением различных звеньев лимфопоэза. Под действием кортизола снижается гиперчувствительность организма к различным антигенам.

Однако иммунная система функционирует на границе между здоровьем и болезнью, следовательно, иммунный ответ может быть защитным и адаптивным. Тем не менее когда иммунная система гиперактивна в течение длительного времени, она может вызвать неадаптивные нейроэндокринно-индуцированные метаболические нарушения.

Так, хроническое воспаление у пациентов с аутоиммунными заболеваниями, по всей видимости, коррелирует с измененной функцией ГГН-оси и формированием стойких или транзиторных нарушений в реализации биологических эффектов кортизола, в т.ч. на иммунную систему. Как уже отмечалось, у ГК показана способность как усиливать, так и подавлять функции иммунной системы. Для объяснения этого феномена предложено две теории, первая состоит в том, что физиология действия ГК соответствует двухфазной кривой зависимости доза–реакция так, что ГК оказывают «разрешающие» (иммуностимулирующие) эффекты в низких концентрациях и подавляющие эффекты в высоких концентрациях. Вторая теория, по сути, продолжает первую и заключается в том, что сеть клеточных эффектов конкурентной ГК-опосредованной «up-регуляции» цитокиновых рецепторов и «down-регуляции» соответствующих цитокинов является биологическим ответом с более быстрым началом, но короткой продолжительностью. Вероятно, в отсутствие любого типа воспаления базальные уровни сигнальных путей рецепторов ГК, которые управляются циркадными ритмами продукции ГК, сенситизируют клетки к повреждающим стимулам путем усиления экспрессии рецепторов цитокинов и факторов комплемента, что позволяет быстро индуцировать воспалительный ответ на повреждение ткани. Во время воспаления, однако, вызванные стрессом концентрации ГК сдерживают иммунный ответ в первую очередь путем ограничения распространения сигналов цитокинов, сокращая таким образом продолжительность иммунного ответа [12][13].

В настоящее время доказано, что при выходе продукции цитокинов и других агентов иммунной системы из-под контроля эндогенных ингибиторов (в том числе стероидных гормонов) эти медиаторы прямо или косвенно становятся ответственными за повреждение тканей и функциональную недостаточность органов, а также гиперкатаболизм. Некоторые медиаторы, высвобождающиеся во время иммунного ответа, а также вызванные ими нейроэндокринные и метаболические реакции могут приводить к повреждению тканей и апоптозу. Клинические и экспериментальные данные указывают на то, что иммунный ответ является защитным, когда он может быстро обезвредить патоген, а данный процесс нуждается в сильной метаболической поддержке [14] и регулировке основных гомеостатических механизмов [15]. Однако, как уже упоминалось, некоторые филогенетически старые и хорошо сохранившиеся продукты иммунных клеток могут опосредовать антигомеостатические реакции.

Антигомеостатические реакции могут наблюдаться при неинфекционных воспалительных/аутоиммунных патологиях, которые в естественных условиях и на поздних стадиях могут быть связаны, в том числе, с нарушениями питания и эндокринными расстройствами. Нейтрализация отдельных цитокинов, которые могут опосредовать такие эффекты, пока невозможна, поскольку цитокины «не работают индивидуально», как правило, вызывают длительные нейроэндокринные и метаболические нарушения. Эволюционно сохраненные антигомеостатические механизмы, характерные для конкретных заболеваний, — предмет дальнейших исследований, результаты которых могут быть очень важны для разработки терапевтических стратегий, которые препятствовали бы нежелательным комбинированным эффектам иммунных и нейроэндокринных медиаторов.
